# Inefficient TLR4/MD-2 Heterotetramerization by Monophosphoryl Lipid A

**DOI:** 10.1371/journal.pone.0062622

**Published:** 2013-04-26

**Authors:** Carolyn R. Casella, Thomas C. Mitchell

**Affiliations:** 1 Institute for Cellular Therapeutics, University of Louisville School of Medicine, Louisville, Kentucky, United States of America; 2 Department of Microbiology and Immunology, University of Louisville School of Medicine, Louisville, Kentucky, United States of America; National Jewish Health and University of Colorado School of Medicine, United States of America

## Abstract

Synthetic forms of *E. coli* monophosphoryl lipid A (sMLA) weakly activate the MyD88 (myeloid differentiation primary response protein) branch of the bifurcated TLR4 (Toll-like receptor 4) signaling pathway, in contrast to diphosphoryl lipid A (sDLA), which is a strong activator of both branches of TLR4. sMLA’s weak MyD88 signaling activity is apparent downstream of TLR4/MyD88 signaling as we show that sMLA, unlike sDLA, is unable to efficiently recruit the TNF receptor-associated factor 6 (TRAF6) to the Interleukin-1 receptor-associated kinase 1 (IRAK1). This reduced recruitment of TRAF6 explains MLA’s lower MAPK (Mitogen Activated Protein Kinase) and NF-κB activity. As further tests of sMLA’s ability to activate TLR4/Myeloid differentiation factor 2 (MD-2), we used the antibody MTS510 as an indicator for TLR4/MD-2 heterotetramer formation. Staining patterns with this antibody indicated that sMLA does not effectively drive heterotetramerization of TLR4/MD-2 when compared to sDLA. However, a F126A mutant of MD-2, which allows lipid A binding but interferes with TLR4/MD-2 heterotetramerization, revealed that while sMLA is unable to efficiently form TLR4/MD-2 heterotetramers, it still needs heterotetramer formation for the full extent of signaling it is able to achieve. Monophosphoryl lipid A’s weak ability to form TLR4/MD-2 heterotetramers was not restricted to synthetic *E. coli* type because cells exposed to a biological preparation of *S. minnesota* monophosphoryl lipid A (MPLA) also showed reduced TLR4/MD-2 heterotetramer formation. The low potency with which sMLA and MPLA drive heterotetramerization of TLR4/MD-2 contributes to their weak MyD88 signaling activities.

## Introduction

New vaccine development by pharmaceutical companies is focused on non-infectious subunit vaccines, but the increase in safety seen with these vaccines sacrifices the usefulness of naturally occurring adjuvant compounds such as bacterial cell wall components and nucleic acids normally present in attenuated or whole killed vaccine preparations [1], [2]. Thus much attention has been focused on identifying adjuvants that can make subunit vaccines more efficacious while having few side effects. The first adjuvant approved by the FDA since alum is a modified bacterial cell wall component, monophosporyl lipid A, MPL adjuvant™. Although MPL adjuvant™ is made from the endotoxin lipopolysaccharide (LPS), removal of the 1 phosphate from the diphosphoryl active component of endotoxin, lipid A, renders it >2,000 fold less toxic in rabbits [3]. We reported previously that a generic form of monophosporyl lipid A, MPLA, is a potent activator of T cell expansion [4], [5] and while MPLA activates the same Toll-like receptor as LPS, it does not activate the same level of proinflammatory cytokines [4], [6]–[11]. We have long been interested in discovering the molecular mechanism of MPLA’s low toxicity because understanding it will help with rational design of new classes of adjuvants such as next generation mimetics of MPLA.

LPS and its derivatives, lipid A and MPLA are recognized by the TLR4/MD-2 complex [9], [12]–[15]. LPS binding protein (LBP) captures monomers of LPS from the cell walls of bacteria or from aggregates in the blood and transfers them to CD14. CD14 in turn transfers LPS to the TLR4/MD-2 complex such that up to 5 acyl chains of LPS sit in the hydrophobic pocket of MD-2 [16], [17]. The crystal structure shows that the TLR4/MD-2/LPS complexes form higher order structures in which LPS’s acyl chains outside of the MD-2 pocket along with its phosphate groups contribute to the interaction between one TLR4/MD-2 and another [17]–[19]. For the hexa-acylated form of LPS the 1 and 4′ phosphates on its diglucosamine head group interact with the TLR4 partnered with the binding MD-2 and the 1 phosphate also interacts with the dimer interface [17]. It is interesting to note that MPLA lacks the 1-phosphate that appears to interact with the dimer interface. Several mutations in MD-2 reveal amino acid residues that seem crucial for TLR4/MD-2 heterotetramer formation [16], [20]. One such mutation is the phenylalanine F126 on MD-2. Mutation of this residue to alanine prevents the formation of higher order TLR4/MD-2 complexes while allowing normal binding of Lipid A, TLR4 and CD14 [16], [17], [19]–[21].

Four adaptor proteins are associated with TLR signaling, Mal (MyD88 adaptor-like protein), MyD88, TRAM (TRIF-related adaptor molecule), and TRIF (Toll IL-1 receptor domain-containing adaptor-inducing IFNβ) [22]–[27]. All of the TLR’s utilize the MyD88 adaptor protein with the exception of TLR3 which requires only the adaptor TRIF. TLR4 is unique in that it uses all 4 signaling adaptors and its signaling events are often divided into MyD88 dependent and TRIF dependent events [22]. After LPS is bound to TLR4/MD-2, MAL and MyD88 are recruited to the Toll/Interleukin-1 receptor (TIR) domain in the cytoplasmic region of TLR4 through PIP2 (phosphatidylinositol 4,5-bisphosphate) interactions [28]. Death domain containing Interleukin-1 receptor-associated kinases (IRAK)1/2/4 are then recruited to MyD88’s death domain and a higher order structure called the myddosome is formed [29], [30]. The IRAKs auto and cross phosphorylate each other leading to recruitment of TNF receptor-associated factor 6 (TRAF6) and ubiquitination of IRAK1 [31]. TRAF6 is also ubiquitinated and recruits TAK-1 (TGFβ activated kinase 1) leading to MAP kinase (MAPK) and nuclear factor-κB (NF-κB) activation [31]–[34]. This MyD88 pathway leads to strong transcriptional activation of proinflammatory cytokines [35]–[37].

TLR4, as mentioned above, also employs the TRIF accessory branch of the TLR signaling pathways. This signaling branch appears to require CD14 for internalization of TLR4/MD-2/LPS into an endosomal compartment in which TRAM can interact with the TIR domain of TLR4 and recruit TRIF [28], [38], [39]. Signaling via the TRIF/TRAM branch does not recruit or activate IRAK but does recruit TRAF6 leading to TAK1 and RIP1 (receptor interacting protein 1) activation [40], [41]. This in turn activates MAPK and NF-κB, albeit with kinetics that is slightly delayed relative to those of the MyD88 pathway [25], [26]. Unlike MyD88, TRIF recruits TNF receptor-associated factor 3 (TRAF3) which, through TANK-binding Kinase 1 (TBK1), causes the phosphorylation and dimerization of interferon regulatory factor 3 (IRF3) [42], [43]. This activation of IRF3 allows genes with an interferon response element to be transcribed; TRIF is therefore referred to as the Type-1 interferon pathway. However, it is an oversimplification to state that MyD88 leads to proinflammatory cytokines while TRIF activation leads to type-1 interferon production. For TLR4, engagement of both pathways is necessary for full activation of proinflammatory cytokines as cells deficient in TRIF are drastically reduced in IL6 (interleukin 6), TNFα (tumor necrosis factor alpha), and IL12p40 (interleukin 12 p40 subunit) production [24]–[26].

Understanding how beneficial immune responses can be generated with the low toxicity monophosporyl form of lipid A, requires some knowledge of the signaling difference from its endotoxic counterpart, lipid A. We have previously reported that the *S. minnesota* biological form, MPLA, and the synthetic *E. coli* like form designated here as sMLA are both weak at activating the MyD88 branch of the TLR4 pathway [4], [7], [8], [44] in comparison to their diphosphorylated counterparts. We now postulate that the lack of one phosphate group could alter interactions needed for the TLR4/MD-2 heterotetramerization such that weak MyD88 signaling results. In order to determine if sMLA is weaker than sDLA in TLR4/MD-2 heterotetramer formation we employed the use of the monoclonal antibody MTS510. The MTS510 mAb binds TLR4/MD-2 heterodimers on unstimulated cells, but not when the cells are exposed to agonist at concentrations that can drive TLR4-TLR4 interactions, as detected in pull down experiments [20], [45]–[47]. Further, MTS510 can bind TLR4/MD-2 when incubated with concentrations of the TLR4 antagonists E5531, that is able to block binding and thus signaling of LPS, but unable to drive TLR4-TLR4 interactions [46], [48]. Thus it appears that MTS510 binds an epitope on TLR4/MD-2 that is obscured upon its heterotetramerization. Using MTS510 mAb staining as an indication of heterotetramerization of TLR4/MD-2, we found that sMLA is indeed weaker at driving TLR4/MD-2 heterotetramerization but does require it for its full, albeit weak, MyD88 response.

## Materials and Methods

### Mice and Reagents

C57BL/6 mice and *tlr2*−/− (B6.129-*Tlr2^tm1kir^*/J) mice were purchased from The Jackson Laboratory and kept in a pathogen-free barrier facility in the University of Louisville. All care and experiments regarding mice were approved by the Institutional Animal Care and Use Committee at the University of Louisville (protocol numbers: 10004 and 10005). Synthetic *E. coli* monophosporyl lipid A (sMLA) was produced by Avanti Polar Lipids, MPLA (PHAD™). Synthetic *E. coli* lipid A (sDLA) was purchased from Peptides International. Synthetic forms were dissolved in DMSO and small aliquots frozen −80°C until use. Mono and Di-phosphoryl lipid A from *S. minnesota* Re595 (MPLA and Lipid A, respectively) were purchased from Alexis-Enzo Life Sciences, sonicated 1hour in a 60°C water bath and stored at 4^o^ prior to use. Alexis-Enzo Life Sciences MPLA and Lipid A are tested by the manufacture on TLR4 deficient mouse splenocytes and macrophages and do not have TLR2 activity.

### Cell Culture

Bone marrow-derived dendritic cells (BMDC) were prepared following the protocol of Lutz *et al*. [49]. Briefly bone marrow from the femurs and tibiae of C57BL/6 or *tlr2*−/− mice were washed in HBSS and cultured on 100 mm bacterial plates in batches of 2×10^6^ BM cells per plate in R10F (RPMI 1640 medium containing, 2 mM L-glutamine, 50 units/ml penicillin, 50 µg/ml streptomycin, 1 mM sodium pyruvate, and 10% heat-inactivated FBS (Valley Biomedical)) with the addition of fresh 50 µM 2-mercaptoethonal and 5 ng/ml recombinant mouse GMCSF (R&D systems). Cells were fed on days 3, 6, and 8 and non-adherent cells were collected on day 9–11. Cells generated by this method were 85–95% CD11b^+^, CD11c^+^, CD86^low^, GR1^−^, CD8^−^, CD4^−^, B220^−^, CD19^−^.

HEK293 cells were purchased from ATCC (CRL–1573) and cultured in High Glucose DMEM containing 1 mM sodium pyruvate, 2 mM L-glutamine, 50 units/ml penicillin, 50 µg/ml streptomycin, and 10% heat inactivated FBS. Cells were transfected with the expression vector pDuo mMD-2/CD14 or a mutated pDuo m(F125AMD-2)/CD14 using Lyo Vec™ (Invivogen). The F126A mutation in MD-2 was created using PCR and the final clone was sequenced to ensure that only the introduced mutation was present. Cell clones were screened by flow cytometry for the presence of the co-expressed CD14 protein and maintained in 100 µg/ml HygroGold™ (Invivogen).

### Immunoblots and Immunoprecipitation

BMDC were seeded in 12×75 mm polystyrene tubes at 2–3 or 3–5×10^6^ cells/ml for immunoblots or immunoprecipitation, respectively. Cells were rested 2 hours at 37^o^ C and then activated with sMLA or sDLA at a concentration of 100 ng/ml for indicated times. For immunoblots cells were washed once with ice cold HBSS containing 50 µM NaF and then lysed in RIPA buffer (50 mM Tris-HCl pH7.4, 150 mM NaCl, 1 mM EDTA, 1% Triton x-100, 1% sodium deoxycholate, 0.1% SDS) containing phosphatase inhibitor mixture (Sigma) and Complete mini protease inhibitor mixture tablets (Roche Applied Science). Protein in clarified lysates was quantified using the Pierce BCA kit and equal amounts of protein were separated on a SDS-PAGE reducing gel. Gels were transferred to nitrocellulose and probed for indicated proteins. To confirm equal loading, blots were either cut in half or stripped and probed for β-actin or stripped and probed for the unphosphorylated protein. For immunoprecipitation cells were lysed in 50 mM Tris HCl pH 7.4, 100 mM NaCl, 1 mM EDTA, 50 mM NaF, 2X complete mini protease inhibitor mixture (Roche Applied Science), phosphatase inhibitor mixture (Sigma) and either 1% Digitonin or 0.2% NP-40. The lysate was pre-cleared with either Protein A/G or mouse IgG-agarose conjugate. Equal amounts of protein as determined by BCA assay were incubated with IRAK-1 antibody or IRAK-1antibody- agarose conjugate overnight and the IRAK-1 antibody incubated lysates were further incubated with Protein A/G (Santa Cruz Biotechnology). Samples were washed and resuspended in 2X protein loading buffer, heated and loaded on a SDS-PAGE gel. Blots prepared from these gels were probed for TRAF 6 and either cut or stripped and re-probed for IRAK-1. Antibodies IRAK-1 F-4 sc-5288, IRKA-1 F-4 AC sc-5288AC, β-actin sc1616 were all from Santa Cruz Biotechnology. TRAF 6 ab 33915 was from Abcam. The antibodies to phospho-ERK 1/2 #4370, ERK1/2 # 4695, IκBα #4814, phospho-IKKαβ #2078 were from Cell Signaling Technology. Secondary antibodies conjugated to horseradish peroxidase (HRP) were purchased from Jackson ImmunoResearcch Laboratories, Inc. After secondary antibody incubation immunoblots were incubated with ECL Plus (GE Healthcare) followed by visualization of the bands with either film or the FujiFilm LAS-4000(mini) Luminescent Image Analyzer. Band intensities were determined using the Quantity One 4.6.6 software (Bio-Rad) for film and Multi Gauge software (FujiFilm) for blots imaged on the LAS-4000(mini).

### Cell Staining

BMDC derived from C57BL/6 mice (for sMLA and sDLA), or C57BL/6 and *tlr2*−/− mice (for MPLA and Lipid A) were incubated with sMLA, sDLA, MPLA or Lipid A at indicated concentrations for 15 min. at 37°C. Cells were then put on ice, washed with ice cold Staining Buffer (SB; 2% Heat Inactivated FBS plus 0.02% NaN_3_ in HBSS) and blocked with SB plus 10% normal rat serum and 20% Fc Block for 10–30 min. on ice. Cells were then incubated with CD11c APC (BD Pharmingen) and one of the following eBiosciences antibodies: MTS510 PE #12-9924, Rat IgG2a K isotype control PE #12-4321, UT41 #12-9041, or Mouse IgG1K isotype control PE #12-4714 for 30 min on ice. Cells were washed with ice cold SB 2 times and kept on ice until analyzed on a FACSCalibur, BD Bioscience. HEK293 cells were transfected with pMIT (an expression vector that has an IRES thy1.1 cassette) and pUNOmTLR4HA3x (Invivogen) or pMIT-MD-2 (an expression vector that contains mouse *md-2* IRES *thy1.1* cassette) and pUNOmTLR4HA3x (Invivogen). Expression of TLR4/MD-2 on the surface of the cell was determined by staining transfected cells with MTS510 PE and transfection efficiency by THY1.1 FITC #11-0900 eBioscience.

### ELISA

BMDC were plated at 1×10^5^ cells per well in a 96 well plate and activated with the indicated concentration of sMLA or sDLA. For ELISA’s on MPLA and Lipid A one experiment was performed with TLR2 knockout cells to confirm no contaminating TLR2 activity. The BD OptEIA™ mouse IL-6 ELISA kit (BD Bioscience) was used to determine the amount of IL-6 in the cell supernatant after 6 hours or overnight incubation.

### NF-κB Measurements

HEK 293 cells expressing mouse MD-2/CD14 or F126A MD-2/CD14 were transfected with pUNOmTLR4HA3x (Invivogen) and pNiFty2-SEAP (Invivogen) using LyoVec™ (Invivogen). Transfection efficiencies were determined by staining the cells for TLR4 expression with UT41 or MTS510 mAbs. The pNiFty-2-SEAP reporter plasmid contains 5 NF-κB transcription binding sites and secretes alkaline phosphatase (SEAP) upon promoter activation. Transfected cells were incubated with the indicated amounts of agonist overnight and supernatants were tested for the expression of SEAP using a QUANTI-Blue™ (Invivogen) colorimetric assay read on a Molecular Devices Emax precision microplate reader. EC50 measurements were determined using GraphPad Prism software to perform non-linear regression and log (agonist) vs. response-Variable slope (four parameters) tests.

### shRNA Knockdown

HEK 293 cells expressing mouse wild type or F126A MD-2 and CD14 were transfected with pUNOmTLR4HA3x, pNiFty-2-SEAP and either psiRNA-LucGL3 (as a negative control) or psiRNA-hMyD88, all from Invivogen. Transfected cells were counted after 48 hours and split into three parts. One part was plated at 1×10^5^ cells per well in 96 well plate, activated with 1 µg/ml sMLA or sDLA for F126A MD-2 cells, or 100 ng/ml for wild type MD-2 cells. Culture supernatants were collected after overnight incubation and the presence of SEAP was measured as an indication of NF-κB activity. The second part was stained for TLR4 expression with the MTS510 mAb and analyzed by flow cytometry. The shRNA plasmids from Invivogen co-express GFP enabling both TLR4 and shRNA expression to be measured in transfected cells. The third part was used to isolate RNA using the Quiagen RNeasy Plus kit. 500 ng of RNA was used in the Quanta qScript cDNA Synthesis Kit to make cDNA. The human GAPDH and MyD88 primers from QuantiTect were used in Quantitative real-time Polymerase chain reaction (RT-PCR) with SYBR Green Master Mix (Applied Biosystems). RT-PCR was performed on a CFX96 Real-Time System C1000 Thermal Cycler (Bio-Rad). The relative amount of MyD88 mRNA per sample was calculated using the comparative cycle method (ΔΔC_t_).

### Statistical Analysis

GraphPad Prism software was used to perform the statistical tests indicated in the figure legends. These include, non-linear regression, log (agonist) vs. response-Variable slope (four parameters) analysis, comparison of fits on Log EC50 using the Extra sum-of squares F test, two way ANOVA with Sidak’s multiple comparisons or Tukey’s multiple comparison test, and two-tailed paired and unpaired T tests.

## Results

### Synthetic Monophosporyl Lipid A is Reduced in its Ability to Stimulate Recruitment of TRAF6 to IRAK1

**Figure 1:**
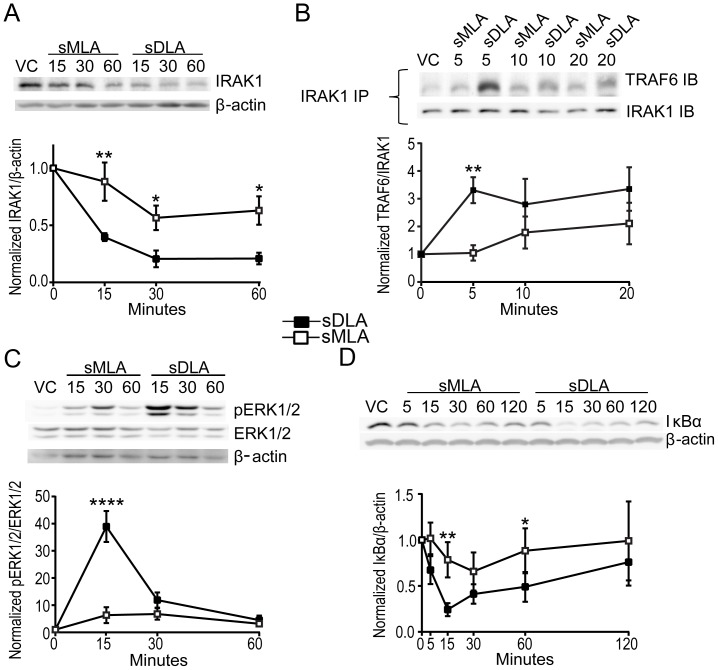
Reduced MyD88-associated signaling by sMLA. Mouse BMDC were treated with 100 ng/ml sMLA or sDLA for the indicated times, lysed and either immunoblots (A,C) or immunoprecipitation (B) were performed on the lysates. A) Immunoblots were probed for IRAK1 and β-actin. A representative gel is depicted with a graph that shows the mean +/− SEM from 5 experiments in which IRAK1 levels were normalized to β-actin levels and vehicle control (VC, measured at 15 min.). B) Lysates were immunoprecipitated with IRAK1 antibodies and immunoblotted with antibodies for TRAF6 and IRAK1. The graph shows mean +/− SEM from 8 experiments with at least 5 data points per time point. TRAF6 levels were normalized based on IRAK1 levels and VC (5 min. time point). C) Left, Immunoblots were probed for phosphoERK 1/2 (Thr202/Tyr204), stripped and re-probed for ERK total, then β-actin. Shown are the mean +/− SEM from 5 experiments with levels of pERK1/2 normalized to ERK total and VC (15 min. time point). Right, immunoblots were probed for IκBα stripped and probed for β-actin. Shown are the mean +/− SEM from 4 experiments with levels of IκBα normalized to β-actin and VC (5 min. time point). Two way ANOVAs with Sidak’s multiple comparisons were performed for A and C and two tailed paired T test for B. Asterisks indicate a significant differences between sMLA and sDLA at the indicated time points with * p<0.05, ** p<0.01 and, **** p<0.001, respectively.

We recently reported that sMLA is reduced in its ability to stimulate ubiquitination of IRAK1 as seen by the disappearance of IRAK1 from immunoblots upon TLR4 engagement [44] and Fig. 1A. To expand on the mechanism of weak MyD88 signaling from sMLA we sought to determine the level of recruitment of TRAF6 to the MyD88 branch of the TLR4 signaling complex because TRAF6 is necessary for strong downstream signaling via MAPK and NF-κB [32], [33]. Figure 1B shows that sDLA can most strongly recruit TRAF6 to IRAK1 within 5 minutes, while sMLA produced low TRAF6 recruitment at all time points tested. sMLA’s weak TRAF6 recruitment activity, in addition to weak involvement of IRAK1 (Fig. 1A), demonstrates its inability to strongly activate early signaling in the MyD88 pathway.

TRAF6 is necessary for the recruitment of TAK1 which in turn leads to activation of MAPK and NF-κB [32], [33]. As shown in Figure 1C and D, sMLA drives weaker MAPK and NF-κB activation as demonstrated by lower phospho-activation of ERK1/2 and weaker degradation of the NF-κB inhibitor, IκBα. Since both TRIF and MyD88 can contribute to activation of MAPKs and NF-κB, it is unclear what level of sMLA’s residual activity is due to weak MyD88 signaling versus what is due to TRIF signaling. However, it is clear from reduced TRAF6 engagement by IRAK1 that MyD88 early signaling events are weaker in sMLA.

### Heterotetramer Formation is Weaker with Synthetic Versions of Lipid A that Lack a Phosphate Group

**Figure 2:**
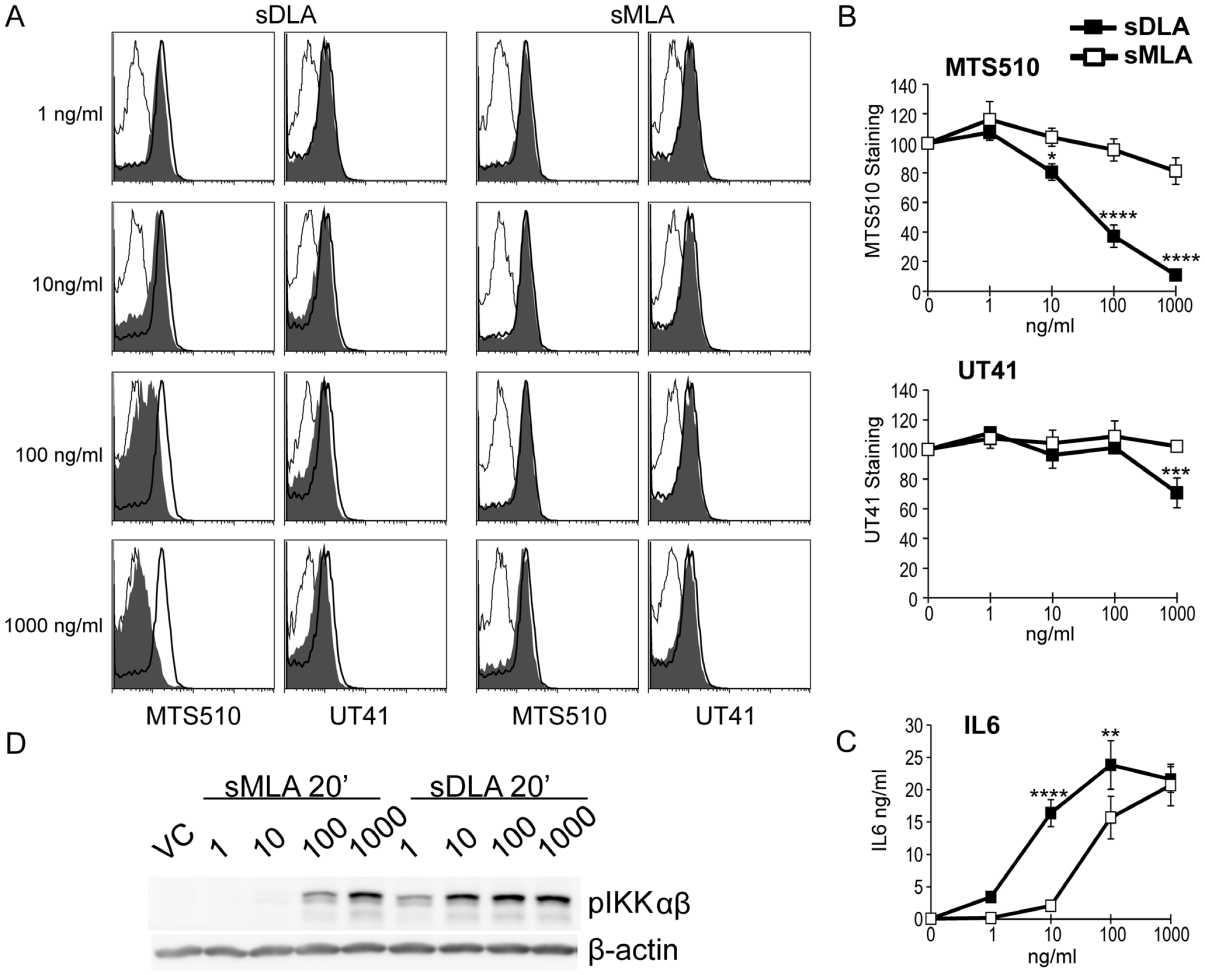
Reduced formation of TLR4/MD-2 heterotetramers by sMLA. A, B) BMDC were incubated with sMLA or sDLA at the indicated concentrations for 15 min. at 37^0^ C. Cells were then stained with MTS510 or UT41 mAb or the appropriate isotype controls along with anti-CD11c. Histograms shown are gated through live CD11c^+^ cells. The histogram overlay is of agonist activated cells stained with MTS510 or UT41 mAb (gray filled), or isotype mAb (light line) and vehicle control treated cells stained with MTS510 or UT41 mAb (dark line). B) The geometric mean signals from all antibody stains was used in the equation (((VC MTS510-VC isotype)-(agonist MTS510-agonist isotype))/(VC MTS510-VC isotype)) X 100 to calculate staining intensities for each dose indicated. The same formula was used for UT41 mAb stains. The graphs show the mean +/− SEM from 7 experiments (1–10 ng/ml) or 8 experiments (100–1,000 ng/ml) for MTS510 mAb staining and 6 experiments for UT41 mAb staining. C) Immunoblots of BMDC activated with 1–1000 ng/ml of sMLA or sDLA for 20 min. and probed for phospho-IKKα(Ser176)/IKKβ(Ser177). The blot shown is representative of one of three similar experiments. D) Using the same cell cultures as A and B, IL-6 ELISA was performed and the mean of 5 experiments with triplicate samples +/− SEM is shown. The asterisk represents, for B a significant difference from vehicle control, and for D a significant difference between sDLA and sMLA, with *p<0.05, **p<0.01, ***p<0.001, and **** p<0.0001, respectively. A two-tailed paired T test was performed for the MTS510 measurements, and a two-way ANOVA with Sidak’s multiple comparisons was performed for the rest.

Solution of the crystal structure of TLR4/MD-2 bound to LPS revealed a role for sMLA’s missing phosphate in the TLR4/MD-2’s heterotetramer interface [17]. Failure to heterotetramerize TLR4/MD-2 leads to weak MyD88 signaling [20], [45], as is seen for sMLA (Fig. 1). These observations led us to hypothesize that sMLA is either unable to heterotetramerize TLR4/MD-2 or much less potent at doing so. Because we were interested in understanding sMLA’s action in primary mouse BMDC as a model of immunostimulatory effects, we decided to make use of the TLR4/MD-2 specific antibody MTS510. When LPS binds TLR4/MD-2, under conditions favorable for heterotetramer formation, MTS510 loses the ability to bind TLR4/MD-2 [20], [45]–[47]. Hence, prevention of MTS510 binding can be used as an indicator of TLR4/MD-2 heterotetramer formation. We incubated BMDC with increasing amounts of sMLA or sDLA for 15 min., at 37°C and then stained the cells with pre-conjugated MTS510 mAb. As a control for loss of TLR4 due to internalization mAb UT41, which detects surface TLR4 without regard to its dimerization status, was also used. Figure 2A and B show that as expected the potent sDLA was able to reduce the staining of MTS510 in a dose dependent manner. At this time point, reduced staining was not due to receptor internalization because UT41 mAb remained able to bind TLR4 on the surface with only a slight decrease in staining at the highest dose. By contrast, sMLA showed no statistically significant reduction in either MTS510 or UT41 staining although at the highest dose tested, 1 µg/ml, sMLA approached significance (p value 0.07) with a small (19%) reduction in MTS510 staining.

In order to correlate heterotetramer formation with signaling outcomes we measured levels of secreted IL-6 in parallel to MTS510 staining. As seen in Figure 2C, 1000 ng/ml sMLA induced high levels of IL6 which was correlated with strong activation of the NF-κB activating kinases, IKKαβ (Fig. 2D). However, doses at which sMLA (1,000 ng/ml) and sDLA (10 ng/ml) induced IL-6 to high levels (Fig. 2C) did not markedly reduce MTS510 staining (Fig. 2A, B). This could be because those doses, while sufficient for strong IL-6 induction, did not achieve the threshold of TLR4/MD-2 heterotetramerization needed to see a reduction in MTS510 staining. Alternatively, a large number of weakly signaling TLR4/MD-2 not in higher order structures was able to produce strongly integrated signals. Thus, we cannot conclude that sMLA is absent in heterotetramerization only that it is much reduced in comparison to sDLA.

### Heterotetramer Formation is Necessary for both sMLA and sDLA to Achieve Maximal Activation

**Figure 3:**
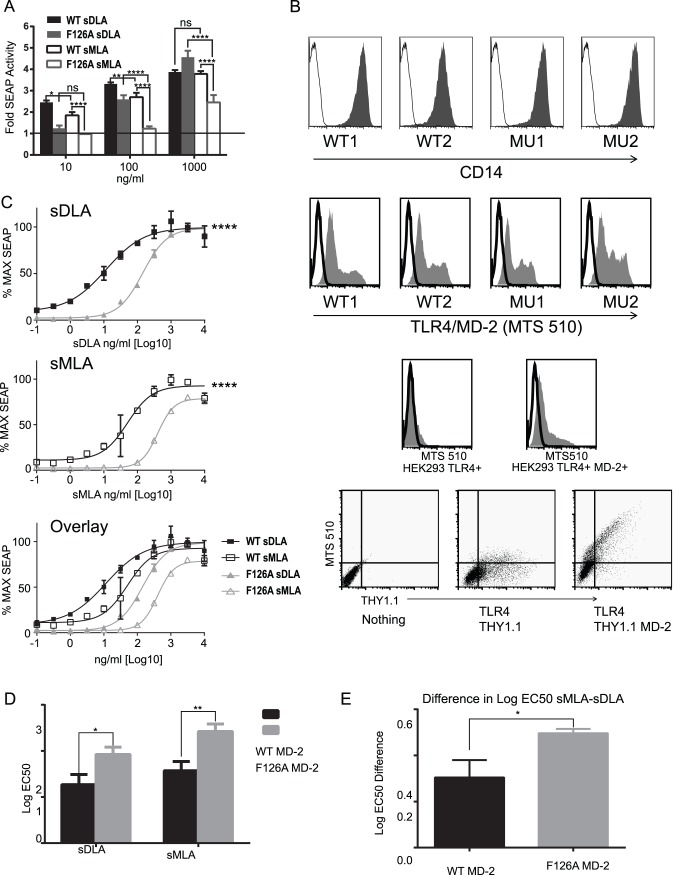
sMLA is further reduced in signaling if heterotetramerization of TLR4/MD-2 is prevented. HEK 293 cells were engineered to express either mouse MD-2 and CD14 or mouse F126A MD-2 and CD14 and transfected with a mouse TLR4 construct and a reporter plasmid that expresses SEAP upon activation of NF-κB. A) Fold-increase in SEAP activity was calculated by dividing the SEAP activity of the agonist by the SEAP activity from the vehicle control for that cell line. The graph shows the mean +/− SEM from 3 experiments in which the TLR4 transfection efficiencies of WT and F126A MD-2 cells were within 10% of each other. B) Histograms and dot blots are of live cells. Two wild type clones (WT) and two F126A MD-2 (MU) clones were stained for the presence of CD14 top panel. Bottom panel staining: HEK293 cells transfected with mouse TLR4 and thy 1.1 expressing construct or TLR4 and a MD-2/thy1.1 expressing construct stained with MTS510. Also shown are the WT1, WT2, MU1, and MU2 clones stained with MTS510 as an indication of surface expression of TLR4 and MD-2. C-E) EC50 measurement. The maximum SEAP values per clone was used to calculate 100% of maximum and a nonlinear curve fit, variable slope with 4 parameters was used to calculate the EC50 of each curve. C) Representative curve fits from one experiment for WT1 and MU2 clones showing the mean +/− SEM. A comparison of fits on Log EC50 using the Extra sum-of squares F test, was performed on the curves. D) Mean values +/− SEM of the log EC50 of three experiments with two clones for WT and MU. E) The difference in the log EC50 for sMLA and sDLA for WT and MU MD-2 as indicated at the bottom of the graph. The experiment in C–E was performed 3 times with triplicate samples for each dose of agonist. Asterisks represent the significant difference between agonist and vehicle control for A, and a significant difference between WT and MU for D and E, with *p<0.05, **p<0.01, ***p<0.001, ****p<0.0001, respectively. A paired two tailed T test was performed for Log EC50 difference, and a two-way ANOVA with Tukey’s multiple comparison test or Sidak’s multiple comparisons were performed A and D, respectively.

In order to determine if sMLA requires TLR4/MD-2 heterotetramer formation for signaling, we made use of the well characterized F126A mutation of MD-2 that is deficient in heterotetramer formation while maintaining normal lipid A, CD14 and TLR4 binding [16], [17], [19]–[21]. If sMLA’s defect in MyD88 signaling is that it cannot drive any heterotetramer formation then two patterns should be observed. First, if sMLA cannot heterotetramerize TLR4/MD-2 then loss of heterotetramerization with the F126A mutation should not affect sMLA’s signaling ability and sMLA should behave the same in cells expressing wild type vs. F126A MD-2 mutant. Second, when heterotetramerization resulting in strong MyD88 activation is absent sMLA and sDLA should be similarly active to one another. We used the HEK293 system to make stable cell lines expressing either mouse wild type (WT) MD-2 or the F126A mutant of MD-2 (MU) and CD14 from the same plasmid. We found it difficult to stably express TLR4 and instead transiently transfected TLR4 along with a NF-κB reporter plasmid expressing secreted alkaline phosphatase (SEAP) that is widely employed as an indicator of TLR4 activation. We noticed that in our TLR4, MD-2, CD14 expressing HEK 293 cells that sMLA appeared to be slightly more potent in comparison to sDLA than in mouse primary BMDC. However, sMLA was still statically less active than sDLA at concentrations of 100 ng/ml and below. We performed a limited dose range experiment with WT and MU expressing cells (Fig. 3A) and are showing only experiments in which TLR4 transfection efficiency of WT and MU cells differed less than 10% in terms of cells transiently expressing TLR4. We found that at low doses of agonist (10 ng/ml), both sDLA and SMLA appeared to be completely dependent on heterotetramer formation as judged by the absence of SEAP made by MU versus WT cells upon exposure to agonist. At the highest dose tested (1,000 ng/ml) sDLA completely, and sMLA partially, overcame this dependence. We also found that sMLA was weaker when heterotetramer formation was prevented by F126A as compared to sMLA in WT cells indicating that sMLA does need heterotetramer formation of TLR4/MD-2 in order to fully signal through MyD88. Also, in mutant F126A cells sMLA is weaker than sDLA indicating that sMLA’s defect cannot be wholly due to lack of efficient heterotetramer formation (Fig. 3A). Hence, neither prediction seemed to be true.

Because clone to clone variation exists, we decided to test 2 WT and MU clones (Fig. 3B) with a more extensive dose range in order to measure EC50 for both sMLA and sDLA in WT and MU cells. We did see some clone variation in expression of the NF-κB reporter plasmid in stimulated cells (not shown). However, sMLA always produced higher EC50 values when MU cells were compared to WT cells in individual experiments. Levels of CD14 surface expression was determined in the clones along with the ability of WT and MU MD-2 to express on the surface of the cell in the presence of TLR4 as determined by the staining of TLR4/MD-2 with the MTS510 mAb which only shows low level TLR4 expression in the absence of MD-2 (Fig. 3B). Overall, both sMLA and sDLA consistently produced higher EC50 values when tested in MU cells that were unable to heterotetramerize their TLR4/MD-2 efficiently (Fig. 3C,D), showing that both relied on heterotetramer competent MD-2 for full potency. In fact, when dose response curves were fitted using the maximal value obtained from each cell line, both sMLA and sDLA reached the same maximal values when tested on wild type cells but not on mutant cells: sMLA was unable to reach the maximal values seen with sDLA even at the highest doses tested (Fig. 3A, C). This pattern is highlighted in Figure 3E which compares the log differences in EC50 from sMLA and sDLA in wild type and mutant cells. Higher values in this graph reflect larger potency differences between sMLA and sDLA on F126A MD-2 MU expressing cells than on WT cells. Thus, sMLA not only appears to be dependent on heterotetramer formation to signal efficiently, but is more dependent on it than sDLA for whatever level of TLR4 signaling it is able to achieve.

### MyD88 Signaling is Involved in NF-κB Activation in Mutant MD-2 Cells

NF-κB activation following TLR4 engagement can occur through both the MyD88 and TRIF signaling branches. Hence, it is possible that the reduction in NF-κB activity we observed when testing the mutant cells was due to the TRIF branch of the signaling pathway only. If this were true, one would expect no difference in signaling outcomes if MyD88 expression were reduced. In order to test this, we used shRNA to knock down expression of MyD88 mRNA in WT and MU HEK 293 cells. We also attempted to use shRNA against TRIF but were unable to knock down more than 10% of its mRNA (data not shown) and thus did not include it in our analysis.

**Figure 4:**
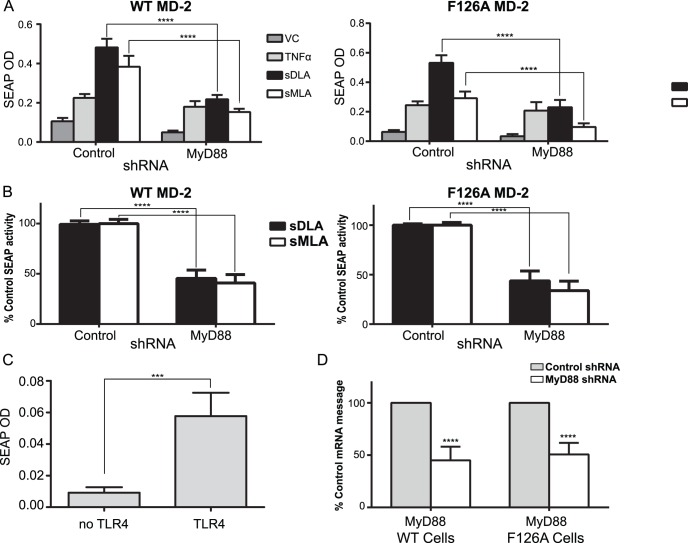
Dependence on MyD88 for NF-κB stimulation in HEK293 cells. HEK 293 cells expressing mouse WT MD-2/WT CD14 or F126A MD-2/WT CD14 cells were transfected with a mouse TLR4 expression plasmid, a NF-κB reporter plasmid expressing SEAP and either LucGL3 control shRNA or MyD88 shRNA. A) Transfected cells were stimulated overnight with vehicle control (VC), 1 ng/ml TNFα, 100 ng/ml sMLA or sDLA for WT and 1 µg/ml for MU cells and SEAP levels were measured. Shown are the means +/− SD from 4 experiments, each with triplicate samples. Measurements of SEAP activity were normalized for TLR4 expression between control and MyD88 shRNA samples. TLR4 expression levels were within 15% of each other. B) Representation of data from (A) where 100% represents SEAP activity after treatment with control shRNA and sMLA or sDLA, respectively. C) WT MD-2/CD14 expressing HEK 293 cells were transfected with NF-κB reporter plasmid and pUNOmTLR4HA3x or pUnoMSC with LucGL3 shRNA. Cells were activated with VC for overnight and SEAP activity was measured. The graph shows the mean +/− SD from 2 experiments with triplicate samples. D) Mean +/− SD of Real Time PCR of MyD88 mRNA levels compared to levels in control shRNA transfected cells, corrected for transfection efficiency of shRNA which was on average 46.5+/−3.3 (SD) and 66.8+/−5 (SD) for WT and MU, respectively. A two-way ANOVA with Sidak’s multiple comparisons was performed for A, B, C; ****p<0.0001 and a two-tailed unpaired t test was performed for C, p = 0.0007.

We tested WT or MU MD-2/CD14 expressing cells, that had been transfected with TLR4, a NF-κB reporter plasmid, and either control or MyD88 shRNA, for NF-κB reporter expression, transfection efficiency, and MyD88 mRNA levels. The vehicle control values differed when cells were expressing either the control or MyD88 shRNA’s and thus raw values are presented rather then fold differences (Fig. 4A) with values expressed as percent of control in Figure 4B. This difference in vehicle control values seemed due to reduction of constitutive TLR4 signaling by the MyD88 shRNA because unactivated WT cells that lacked TLR4 showed lower background NF-κB activity than TLR4^+^ counterparts (Fig. 4C). Over 93% of the cells expressing surface TLR4, and thus the cells responding to agonist, were also transfected with shRNA (data not shown). Further, when corrected for transfection efficiency of shRNA, the MyD88 shRNA was observed to reduce approximately 50% of MyD88 message in MU and WT cells (Fig. 4D). In MD-2 WT cells expression of MyD88 shRNA reduced the NF-κB activity on average 50% for sDLA and 59% for sMLA (Fig. 4B). F126A MD-2 MU cells showed a similar reduction in NF-κB activity when MyD88 shRNA was expressed, 55% for sDLA and 66% for sMLA (Fig. 4B). These results indicate that the majority of TLR4 dependent NF-κB activation for sMLA is through the MyD88 signaling pathway in mouse TLR4 expressing HEK293 cells. And that the NFκB signaling in the mutant cells is not due solely to the TRIF branch. It is interesting to note that in HEK293 cells sMLA appears to activate MyD88 more strongly than in mouse BMDC. While we are not sure why this is the case the high level of TLR4 expressed by transiently transfection of TLR4 could contribute to sMLA’s more potent action in these cells.

### Biological Monophosphoryl Lipid A is also Deficient in Heterotetramerization

**Figure 5:**
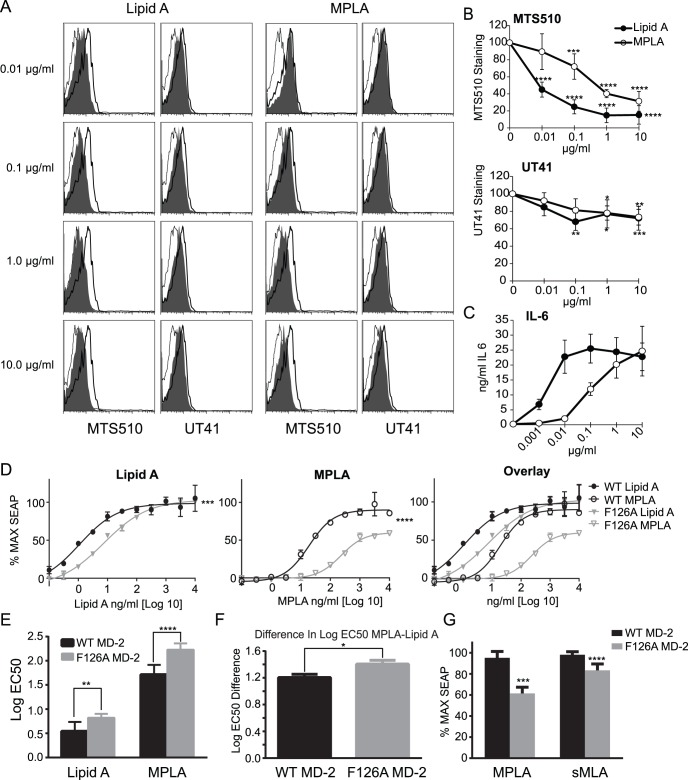
Non-synthetic MPLA also fails to drive heterotetramer formation. A) BMDCs were activated with the indicated concentrations of MPLA or Lipid A for 15 min. at 37^o^ C. The cells were stained with anti-CD11c, and MTS510, UT41, or appropriate isotype controls. Live CD11c^+^ cells were gated and representative histograms are shown. The histogram overlay is of agonist activated cells stained with MTS510 or UT41 mAb (gray filled), or isotype mAb (light line) and vehicle control treated cells stained with MTS510 or UT41 mAb (dark line). B) Mean +/− SD of staining in A from 3 independent experiments as explained in Figure 2, significance represents the difference of sMLA or sDLA from the vehicle control sample. C) Mean +/− SD of IL6 measurements in 2 of the 3 experiments performed in B) with triplicate samples. D-G) EC50 measurement. HEK293 cells expressing either WT or F126A MD-2 and CD14, were transfected with TLR4 and a reporter plasmid that expresses SEAP upon NF-κB activation. Three independent experiments with triplicate samples were performed using two WT and MU cell clones. Maximal values were plotted as 100% to create a dose response curve. GraphPad Prism’s nonlinear curve fit, variable slope with 4 parameters was used to determine EC50’s. C) An example from one experiment of dose response curves used to measure EC50 is shown with mean +/− SD. A comparison of fits on Log EC50 using the Extra sum-of squares F test, was performed on the curves. D-F) The mean of 3 experiments with triplicate samples +/− SEM is depicted. G) The average +/− SEM of % max reached in EC50 measurements for MPLA in above experiments and sMLA in experiments from Fig. 2. A two way ANOVA with Sidak’a multiple comparisons (B, E), a paired two tailed T test (F), and an unpaired two tailed T test (G) was used to test for statistical significance. Asterisks represent, for B a statistical differences between agonist and vehicle control, for E-G statistical differences between WT and MU, *p<0.05, **p<0.01, ***p<0.001, ****p<0.0001, respectively.

Synthetic lipid A preparations are useful in that they are homogenous. However, the vaccine adjuvant MPL ™ is a biological preparation manufactured from *S.minnesota* Re595 cultures and contains a heterogeneous mixture of tetra to hexa acylated forms of monophosporyl lipid A [50]. To model MPL ™ more accurately we decided to use a commercially available, generic, biological preparation of *S. minnesota* monophosporyl lipid A (MPLA) and diphosphoryl lipid A (Lipid A). We again used the MTS510 and UT41 antibodies to determine the extent to which heterotetramers of TLR4/MD-2 form. As seen in Figures 5A and B, MPLA drove heterotetramerization with more potency than sMLA, but there was still a large reduction in the amount of heterotetramer formation compared to Lipid A. The amount of heterotetromers of TLR4/MD-2 observed with the biological preparation seems to correlate more strongly with IL6 production than was observed with the synthetic sMLA and sDLA (Fig. 2B, C; 5B, C).

To confirm that TLR4/MD-2 heterotetramer formation is necessary for MPLA’s function we again determined the EC50 in HEK 293 cells that expressed either WT MD-2 or the F126A mutant of MD-2 along with CD14, TLR4 and a NF-κB reporter plasmid. Both Lipid A and MPLA had higher EC50 values for heterotetramer-deficient MD-2 as compared to wild type, indicating a need for heterotetramer formation for maximum function (Fig. 5D,E). Altered patterns were also observed when comparing sMLA to MPLA because the measured differences in EC50 from their diphosphoryl counterparts were much more pronounced for MPLA than sMLA in WT cells, 1.2 Log and 0.3 Log, respectively (Fig. 2E, and 5F). Furthermore, in the MU cells MPLA was only able to reach on average 62% of the max of Lipid A whereas sMLA could reach 83% of sDLA’s maximum value (Fig. 5F). Thus, the biological preparation of MPLA appears to be more dependent on heterotetramer formation for maximal activity than is true of the synthetic MLA.

## Discussion

MPLA is known to be a weak activator of proinflammatory cytokines, which require strong MyD88 activation to be maximally expressed [4], [6]–[11]. Discovering why MPLA is weak at activating MyD88 has long been a goal of our laboratory. Here we show in primary mouse BMDC that MPLA and a synthetic version of it, sMLA, are both reduced in their ability to form TLR4/MD-2 heterotetramers. Downstream of this weak heterotetramer formation is low ubiquitination of IRAK1 and recruitment of TRAF6. Weak TRAF6 recruitment after MPLA stimulation is apparent in weak NF-κB and MAPK activation and manifested in turn as and lower proinflammatory cytokine production [4], [7], [8] (Fig. 1C).

We previously reported that while MPLA is unable to strongly activate the MyD88 branch of the TLR4 signaling cascade it is comparatively better at activating TRIF dependent outcomes [4], [7], [8]. Meng J. *et al.* showed, using HEK293 cells, that TLR4 with mutated phosphate binding domains is equally deficient in its ability to signal through the MyD88 and TRIF pathways [51]. From this observation they concluded that MPLA, which is missing one phosphate, should affect both branches of TLR4 in a similar manner. However, nuanced differences in strength of signal might not be apparent when testing engineered HEK293 cells, which are often associated with unnaturally high levels of receptor expression. Although mutation of either phosphate-binding site rendered mouse TLR4 incapable of MyD88 or TRIF-dependent signaling in Meng J. *et al*.’s HEK293 system, we found that MPLA can signal fairly well in HEK293 cells expressing mouse TLR4/MD-2. This leads to the question, why would MPLA, which can’t efficiently heterotetramerize TLR4/MD-2, be able to activate the TRIF branch more robustly in our previous experiments? It is possible that in the endocytic compartment, where TRIF signaling originates, there is a less stringent requirement for formation of TLR4/MD-2 heterotetramers. If so this would result in better TRIF signaling for an agonist that is inefficient at forming heterotetramers of TLR4/MD-2 such as MPLA and sMLA [52]. Or perhaps MPLA is weak at activating both branches as Meng J. *et al*. suggest, but for TRIF dependent products, a lower strength of signal is needed than for MyD88 resulting in apparent better TRIF signaling than MyD88. In fact different strength of signal needed for differing outcomes could explain why sMLA is able to activate SHIP through MyD88 strongly [44] but not other proinflammatory cytokines. It is possible that SHIP activation does not require as strong signaling through TLR4/MD-2 and thus sMLA is able to activated it well while not being able to active other proinflammatory cytokines. We are continuing to work on discovering why MPLA is better able to activate TRIF dependent genes downstream of TLR4.

If the only deficiency in TLR4 engagement by MPLA is that it is unable to drive hetrotetramerization, then one would expect in the F126A MD-2 mutant cells, where heterotetramer formation is undetectable, that MPLA and Lipid A would show equivalent activity. Instead, MPLA appears to be even weaker than Lipid A in cells expressing F126A mutant MD-2 as compared to wild type MD-2. It is possible that MPLA is weaker at binding the TLR4/MD-2 complex and when only weak MyD88 signals are allowed, without heterotetramer formation [20], [45], the weaker binding is more apparent. Or perhaps there are differences in an even higher order arrangement of TLR4/MD-2 complexes beyond heterotetramer formation. It will be important in the future to determine the binding affinity of MPLA and Lipid A for TLR4/MD-2 to fully understand the complex nature of MPLA’s activity.

Biologically-derived preparations of *S. minnesota* lipid A seem to be able to drive heterotetramerization of TLR4/MD-2 at lower concentrations than synthetic *E.coli* lipid A. This seems to be true for both the di- and mono- phosphoryl preparations. These differences could be due to slightly different structures of sMLA (*E.coli*-like) and MPLA (*S. minnesota*), which are similar but not identical, or the fact that MPLA contains tetra- and penta-acylated along as well as the hexa-acylated form [50]. It is interesting to note, that at high concentrations, sMLA was able to signal as strongly as sDLA for some proinflammatory cytokines (Fig 2C) [6] without detectable heterotetramer formation. We speculate that a threshold of heterotetramer formation is needed for strong signaling such that at high concentrations of sMLA and low concentrations of sDLA a sufficient number of TLR4/MD-2 heterotetramers form and fully activate NF-κB and cytokines such as IL6, but that this number is too low to score as decreased binding in the MTS510 mAb staining assay.

Monophosporyl lipid A is a good activator of T cell priming [4], [5] and additions of MPL-adjuvant ™ to vaccines against HBV and HPV results in their increased immunogenicity [9], [53]–[55]. MPLA is able to do this while being a weak activator of MyD88 signaling and thus, a weak activator of proinflammatory cytokines [4], [6]–[8], [10], [44]. Here we show using primary mouse cells that MPLA’s weak MyD88 signaling can be explained by its inability to drive formation of TLR4/MD-2 heterotetramers as efficiently as Lipid A does, which immediately results in lower ubiquitination of IRAK-1 and TRAF6 recruitment. Although MPLA is reduced in heterotetramer activity, it nevertheless requires these higher order structures to signal maximally. A recent study performed with human TLR4 expressed in the mouse BA/F3 cell line came to the same conclusion for human TLR4/MD-2 [56]. MPLA is reduced in the formation of human TLR4/MD-2 heterotetramers but needed to form heterotetramers for full function, showing that this aspect of signaling by MPLA appears to be the same for human and mouse TLR4. Measurement of the extent of heterotetramerization of TLR4/MD-2 by TLR4 agonists could provide a useful screen for next generation vaccine adjuvants.
